# A phase II open label, randomised study of ipilimumab with temozolomide versus temozolomide alone after surgery and chemoradiotherapy in patients with recently diagnosed glioblastoma: the Ipi-Glio trial protocol

**DOI:** 10.1186/s12885-020-6624-y

**Published:** 2020-03-12

**Authors:** Nicholas F. Brown, Stasya M. Ng, Claire Brooks, Tim Coutts, Jane Holmes, Corran Roberts, Leena Elhussein, Peter Hoskin, Tim Maughan, Sarah Blagden, Paul Mulholland

**Affiliations:** 1 Department of Oncology, University College London Hospitals, 250 Euston Road, London, NW1 2PQ UK; 2 Oncology Clinical Trials Office (OCTO), Department of Oncology, The University of Oxford, Old Road Campus Research Building, Oxford, OX3 7DQ UK; 3 Centre for Statistics in Medicine (CSM), University of Oxford, Botnar Research Centre, Windmill Road, Oxford, OX3 7LD UK; 4 Mount Vernon Cancer Centre, Rickmansworth Road, Northwood, HA6 2RN UK; 5 Oxford Institute for Radiation Oncology, University of Oxford, Old Road Campus Research Building, Roosevelt Drive, Oxford, OX3 7DQ UK; 6 Department of Oncology, University of Oxford, Old Road Campus Research Building, Oxford, OX3 7DQ UK; 7 UCL Cancer Institute, 72 Huntley St, London, WC1E 6AG UK

**Keywords:** Glioblastoma, Glioma, Ipilimumab, Temozolomide

## Abstract

**Background:**

Median survival for patients with glioblastoma is less than a year. Standard treatment consists of surgical debulking if feasible followed by temozolomide chemo-radiotherapy. The immune checkpoint inhibitor ipilimumab targets cytotoxic T-lymphocyte-associated protein 4 (CTLA-4) and has shown clinical efficacy in preclinical models of glioblastoma. The aim of this study is to explore the addition of ipilimumab to standard therapy in patients with glioblastoma.

**Methods/design:**

Ipi-Glio is a phase II, open label, randomised study of ipilimumab with temozolomide (Arm A) versus temozolomide alone (Arm B) after surgery and chemoradiotherapy in patients with recently diagnosed glioblastoma. Planned accrual is 120 patients (Arm A: 80, Arm B: 40). Endpoints include overall survival, 18-month survival, 5-year survival, and adverse events. The trial is currently recruiting in seven centres in the United Kingdom.

**Trial registration:**

ISRCTN84434175. Registered 12 November 2018.

## Background

Glioblastoma is the most common malignant primary brain tumour [1]. Survival is poor, with a median survival from diagnosis of 14.6–21.1 months with standard therapy in clinical trials [2–7]. However, registry databases report overall survival of only 6–10 months [8, 9]. Standard therapy is surgical debulking if feasible, with the degree of resection correlating with prognosis [10–13]. This is followed by adjuvant chemoradiotherapy given within 6 weeks of surgery, with 60 Gray (Gy) of fractionated focal external beam radiotherapy administered in 30 fractions over 6 weeks, along with daily concomitant temozolomide 75 mg/m^2^. Following a 28-day break, patients receive six cycles of adjuvant temozolomide 150-200 mg/m^2^, given for 5 days in a 28-day cycle. This standard was implemented following demonstration of a 2.5 month median survival benefit over radiotherapy alone in the landmark EORTC-NCIC randomised phase III trial [6, 14]. There is no standard therapy for patients at relapse who are typically treated with lomustine given as monotherapy or in combination with procarbazine and vincristine (PCV) [15, 16].

The traditional dogma of the CNS as an immune-privileged site has been widely eroded, and there is now convincing evidence that the CNS has a fully functioning, although tightly regulated, innate and adaptive immune system, underpinned by a functional lymphatic system [17]. Malignant gliomas elicit systemic immune dysregulation, with reduced CD4+ T-cell number and function, and increased Tregs [18–21].

The amplitude and quality of T-cell responses are regulated by a balance of co-inhibitory and co-stimulatory signals, termed immune checkpoints. These “checkpoints” allow a rapid and effective response against foreign antigens, whilst preventing overstimulation and auto-immune responses. T-cell activation after antigen recognition by the T-cell receptor is a complex integration of these stimulatory and inhibitory signals. Gliomas exploit these checkpoints, with expression of negative regulators of immune response and conversion of cytotoxic T-cells to regulatory T-cells in the tumour microenvironment to escape immune surveillance [17, 22].

Cytotoxic T-lymphocyte antigen-4 (CTLA-4, CD152) is a CD28 homolog with a 100–1000 higher affinity for B7 (CD80/86). However, unlike CD28, CTLA-4 does not produce a costimulatory signal when bound to B7. The degree of CD28:B7 binding versus CTLA-4:B7 binding determines whether a T cell is activated or undergoes anergy [23]. In resting naïve T cells CTLA-4 is regulated in part by its subcellular localisation: it is principally found in intracellular vesicles and not functional until expressed on the cell surface [24]. CTLA-4 is constitutively expressed on Tregs and plays a key role in generating tolerance [25]. Thus CTLA-4 is a principal regulator of an effective immune response [26–28].

Ipilimumab is a human IgG monoclonal antibody specific for CTLA-4 and blocks the interaction with B7, augmenting T cell activation and proliferation. In preclinical glioblastoma models, systemic CTLA-4 blockade produces an effective T-cell response, tumour shrinkage, and prolongs survival [29–32]. Efficacy in patients with melanoma with brain metastases provide clinical evidence of activity within the central nervous system [33–35].

The aim of this phase II trial is to evaluate the addition of ipilimumab to standard therapy in patients with recently diagnosed glioblastoma. This manuscript describes protocol version 3.0 (13 August 2019).

## Methods

### Study objectives

The primary objective of this study is to evaluate whether the addition of ipilimumab to the current standard of care following surgery and radiotherapy will improve survival in patients with newly diagnosed glioblastoma. Secondary objectives are evaluation of the safety and tolerability of ipilimumab with temozolomide versus temozolomide alone, and whether the addition of ipilimumab to the standard of care improves long term survival. Accordingly, the study endpoints are overall survival, overall survival at 18 months, overall survival at 5 years, and any toxicity grade 3 of higher according to the Common Terminology Criteria for Adverse Events (CTCAE) v4.03 [36].

### Trial design

This study is an open label, stratified randomised, multicentre, phase II clinical trial. Following informed consent, patients will enter a screening phase during which the eligibility for randomisation will be determined. Patients who meet the eligibility criteria will be randomly allocated in a 2:1 ratio to receive either ipilimumab with temozolomide (Arm A) or temozolomide alone (Arm B). A total of 120 patients will be randomised, with 80 patients in Arm A and 40 patients in Arm B. Treatment allocation will be by minimisation incorporating a random element, and will be carried out by computer. Randomisation will be stratified by extent of surgery (total versus subtotal resection) and MGMT promotor methylation (methylated, unmethylated, or unknown). Randomised patients will enter the treatment phase. After stopping study treatment, patients will remain on study for surveillance for 52 weeks following the date of randomisation. Tumour progression will be assessed by contrast enhanced MRI performed every 12 weeks as per standard care until tumour progression. Survival status will be collected at 18 months from the date the last patient is randomised, and at 2, 3 and 5 years from each patient’s randomisation date.

### Patient cohort

Patients are currently being recruited from 7 sites in the United Kingdom (Addenbrooke’s Hospital, Cambridge; Churchill Hospital, Oxford; Guy’s Hospital, London; Mount Vernon Cancer Centre, Middlesex; The Christie, Manchester; University College Hospital, London; Western General Hospital, Edinburgh). The first patient enrolled in January 2019.

Inclusion criteria include: newly diagnosed histologically confirmed de-novo supratentorial glioblastoma with greater than 20% surgical debulking; radiotherapy to have begun within 49 days of surgery; completed standard radiotherapy with concurrent oral temozolomide; age 18–70 years of age; life expectancy of at least 12 weeks; ECOG performance status 0 or 1; haemoglobin ≥9 g/dL; platelet count ≥100 × 10^9^/L; absolute neutrophil count (ANC) ≥ 1 × 10^9^/L; lymphocyte count ≥0.5 × 10^9^/L; serum creatinine < 1.5 upper limit of normal (ULN) or Cockroft-Gault creatinine clearance ≥50 mL/min; bilirubin ≤1.5x ULN (or ≤ 3x if known Gilbert’s syndrome); ALT or AST ≤ 3x ULN.

Exclusion criteria include: multifocal glioblastoma; secondary glioblastoma; known extracranial or leptomeningeal disease; any other treatment for glioblastoma other than surgery and temozolomide chemoradiotherapy; dexamethasone dose > 3 mg daily (or equivalent); significant intra- or peri-tumoral haemorrhage; clinically relevant, active, known or suspected autoimmune disease; history of significant gastrointestinal impairment; history of interstitial lung disease; any condition requiring systemic steroid therapy (> 10 mg prednisolone daily or equivalent); other active malignancy; known history of Hepatitis B or C or HIV; and pregnant or breast-feeding women.

### Study treatment

Study treatments include ipilimumab (Yervoy™) (Arm A) and temozolomide (TMZ) (Arms A and B).

#### Ipilimumab

Ipilimumab will be dosed at 3 mg/kg and administered as an intravenous infusion over 90 min. The first cycle of ipilimumab will be administered within 14 days of completing radiotherapy and within 3 days of randomisation. Ipilimumab will be administered once every 3 weeks for a total of 4 infusions. No dose reduction is permitted.

Dosing will be delayed in the following occurrences: grade ≥ 2 adverse events (AEs) (except grade 2 skin AEs, fatigue, or laboratory abnormalities other than ALT/AST); grade 3 skin AEs; grade 3 laboratory AEs (except grade ≥ 3 lymphopaenia or grade ≥ 3 amylase/lipase if no clinical evidence of pancreatitis). Ipilimumab retreatment may resume once the AE returns to grade ≤ 1 or baseline. Patients may resume treatment with grade 2 fatigue, or drug-related endocrinopathies once adequately controlled with physiological hormone replacement if discussed and approved by the trial management group. Ipilimumab related pulmonary AEs, diarrhoea or colitis must return to baseline prior to resuming treatments except grade ≥ 3 diarrhoea/colitis where ipilimumab must be permanently discontinued. Persistent grade 1 pneumonitis may resume treatment after a steroid taper over at least 1 month.

Ipilimumab will be permanently discontinued if any of the following suspected ipilimumab related AEs occur: grade ≥ 2 uveitis, eye pain, or blurred vision that does not respond to topical therapy and does not improve to grade 1 severity within the retreatment period, or that requires systemic treatment; grade ≥ 3 uveitis, pneumonitis, bronchospasm, hypersensitivity reaction, or infusion reaction; grade ≥ 3 drug-related AEs, with the following exceptions: grade 3 skin AEs, endocrinopathies adequately controlled with only physiological hormonal replacement, and laboratory abnormalities (other than the following which do require discontinuation: grade 3 thrombocytopenia or associated with bleeding, AST or ALT > 5–10 x ULN for > 14 days, AST or ALT > 10 x ULN, total bilirubin > 5 x ULN, concurrent AST or ALT > 3 x ULN and total bilirubin > 2 x ULN); any grade 4 AE except skin AEs only where not considered to be related to ipilimumab, neutropenia, lymphopenia or leukopenia, isolated lipase/amylase elevations without clinical manifestations of pancreatitis and endocrinopathies which resolve or are adequately controlled with physiological hormone replacement (as discussed with trial management group); or dosing delays > 42 days from the previous dose (unless approved by the trial management group).

#### Temozolomide

Temozolomide will be administered orally for 6 cycles in both Arms A & B. Temozolomide will commence as per standard care following completion of chemoradiotherapy. Dosing is once daily for 5 days in a 28-day cycle. The dose in cycle 1 is 150 mg/m^2^ (dose level 0), unless ANC < 1.5 × 10^9^/L, platelet count < 100 × 10^9^/L, or any non-haematological toxicity ≥ grade 2 (except alopecia, nausea, vomiting) during concomitant temozolomide administration with radiotherapy, in which case temozolomide will be initiated at 100 mg/m^2^ (dose level − 1). If no non-haematological toxicity ≥ grade 2 (except alopecia, nausea, or vomiting) is experienced in cycle 1, ANC is ≥1.5 × 10^9^/L, and platelet count ≥100 × 10^9^/L the dose is escalated to 200 mg/m^2^ (dose level 1) from cycle 2 (or to 150 mg/m^2^ if cycle 1 was dosed at 100 mg/m^2^). If the dose was not escalated at Cycle 2, escalation is not done in subsequent cycles. The temozolomide dose will be reduced if ANC < 1.0 × 10^9^/L or platelet count < 75 × 10^9^/L or for grade 3 non-haematological toxicity (except alopecia, nausea, vomiting). If AEs persist, treatment will be delayed by 1 week for up to 4 consecutive weeks, after which if AEs have not resolved to ≤ grade 1 then temozolomide will be discontinued. Temozolomide will be stopped if the same grade 3 non-haematological toxicity (except alopecia, nausea, vomiting) recurs after dose reduction, or if treatment at dose level − 1 results in unacceptable toxicity.

### Statistical considerations

To give a power of 80% to show a significant difference of 22.5 month median survival in Arm A (ipilimumab + temozolomide) and 15 month median survival in Arm B (temozolomide alone) at one sided 20%, allowing for 5% loss to follow-up at 3 years, 120 patients need to be recruited (80 to Arm A, 40 to Arm B). This assumes an 18-month recruitment period and survival follow-up for a minimum of 18 months.

## Discussion

The objective of this study is to evaluate whether the addition of ipilimumab to temozolomide following standard chemoradiotherapy improves survival in patient with glioblastoma. Given the recent failures of phase 3 trials of new therapies in glioblastoma to demonstrate survival benefit following apparent efficacy in single arm phase II trials [37], Ipi-Glio includes a standard of care arm (Arm B), with a 2:1 randomisation between Arm A and B in order to aid recruitment.

A limitation of this study is a lack of biomarkers to provide potential determinants of clinical response to ipilimumab. There is no accepted biomarker for response to ipilimumab therapy. Studies in melanoma have found a number of markers that are associated with response to ipilimumab, including expression of genes associated with antigen presentation [38], the T-cell receptor repertoire [39], HLA-I heterozygosity [40], tumour mutational burden [39], somatic copy number mutation burden [41]; systemic immune response factors such as serum IL-6 levels [42]; and gut microbiome variants [43]. These factors will need to be considered in future studies. Further, antibiotic use in the month prior to administration of the PD-1 checkpoint inhibitor nivolumab is associated with poorer survival [44]. This is of particular importance for glioblastoma, as co-trimoxazole is routinely administered with temozolomide chemoradiotherapy as prophylaxis against P.*jiroveci* pneumonia. In Ipi-Glio, patients are enrolled after completing radiotherapy, but in future trials withdrawal of routine co-trimoxazole should be considered. The Ipi-Glio study is currently ongoing.

## Data Availability

Not applicable.

## Abbreviations

AE: Adverse event
ANC: Absolute neutrophil count
CNS: Central nervous system
CTCAE: Common Terminology Criteria for Adverse Events
CTLA-4: Cytotoxic T-lymphocyte-associated protein 4
Gy: Gray
PCV: Procarbazine, lomustine, vincristine
RT: Radiotherapy
TMZ: Temozolomide
ULN: Upper limit of normal
